# Stem cells isolated from the human stromal limbus possess immunosuppressant properties

**Published:** 2012-07-25

**Authors:** Yonathan Garfias, Jessica Nieves-Hernandez, Mariana Garcia-Mejia, Carlos Estrada-Reyes, Maria Carmen Jimenez-Martinez

**Affiliations:** 1 Research Unit, Institute of Ophthalmology, Conde de Valenciana Foundation, Mexico City, Mexico; 2 Department of Biochemistry, Faculty of Medicine, Universidad Nacional Autónoma de México, Mexico City, Mexico

## Abstract

**Purpose:**

Mesenchymal stromal stem cells (MSC) are non-hemopoietic cells with the capacity to self-renewal and to differentiate into various cell lineages of mesenchymal origin. More recently, the immune regulatory potential of MSC has been focused on. Furthermore, mesenchymal stem cells obtained from diverse tissues possess immunomodulatory properties and inhibit proinflammatory immune reactions. The aim of this study was to determine the immunosuppressive characteristics of mesenchymal stem cells isolated from human limbal (L-MSC) tissue.

**Methods:**

L-MSC were enzymatically obtained from cadaveric sclero-corneal rims and expanded in vitro. The cells were characterized by flow cytometry using specific antibodies to mesenchymal stem cells markers. Clonogenic and tissue transdifferentiation in vitro assays were performed. The effect of L-MSC soluble factors on T cell proliferation was determined by flow cytometry. Cytokines such as transforming growth factor-b1 (TGF-β1) and Interleukin-10 (IL-10) on supernatants from L-MSC were identified by enzyme-linked immunosorbent assay (ELISA).

**Results:**

Herein, we described that L-MSC cells in vitro-expanded were positive for the expression of vimentin, CD29, CD34, CD39, CD73 and CD105 mesenchymal stem cells markers; meanwhile, this cell population was negative to CD45 and HLA-DR hemopoietic markers as well as to cytokeratin expression. Clonogenic assays showed that these cells were able to form colonies. In addition, this L-MSC population had the ability to transdifferentiate into neurons and chondrocytes and to form tubular networks on matrigel in the presence of vascular endothelial growth factor (VEGF). These results indicated that these cells were stem cells. Additionally, soluble factors secreted by L-MSC were capable of mediating the suppression of T-cell receptor (TCR)-engagement lymphocyte proliferation. In an attempt to identify the possible immunosuppressive factors secreted by L-MSC, TGFβ1 and IL-10 cytokines were determined in the L-MSC supernatants by ELISA; interestingly, TGFβ1 was constitutively secreted by this cell population; in contrast, IL-10 was not detectable. Moreover, TGFβRII neutralizing antibodies were able to revert the TCR-engagement lymphocyte proliferation inhibition mediated by L-MSC. Thus, TGFβ1 secreted by L-MSC was able to suppress T cell proliferation.

**Conclusions:**

Taken together these results, explain in part the immunosuppressive features of this cell population obtained from the human limbus. All these characteristics make this cell population an excellent source to be used in the regenerative medicine.

## Introduction

Mesenchymal stromal stem cells (MSC) are non-hemopoietic cells with the capacity to self-renewal and differentiate into various cell lineages of mesenchymal origin [1]. These cells can be obtained from several anatomic sites such as bone marrow, adipose tissue, fetal liver, umbilical cord, amniotic membrane, and from the limbus in the eye [2-6]. More recently, the immune regulatory potential of MSC has been focused on. It has been shown that MSC from bone marrow inhibit lymphocyte proliferation in vitro [7,8], inhibit the production of cytokines [9], as well as the formation of cytotoxic clusters of differentiation (CD8)^+^ T cells [10], and decrease the immune response in vivo [11-14]. Additionally, it has been described that not only the MSC from bone marrow origin have immunoregulatory properties, but skin fibroblasts and MSC from adipose tissue among other cells, have also immunoregulatory properties [15,16].

The junction of the cornea and conjunctiva is known as the limbus, which is used for therapy in patients with limbal stem cell deficiency [17]. There are reports indicating the presence of fibroblast-like cells in the human limbal stroma which possess a stem cell-like self-renewal property [4,18]; and it has recently documented that stem cells obtained from limbal murine tissue possess immunoregulatory properties and inhibit proinflammatory immune reactions [19]. The aim of this study was to determine the immunosuppressive properties of the stromal stem cells isolated from the human limbus.

## Methods

### Reagents and antibodies

Flourescein isothiocyanate (FITC)-labeled antibodies to human vimentin, phycoerythrin (PE)-labeled antibodies to CD39 and FITC-goat anti-mouse secondary antibodies were purchased from Santacruz Biotechnology (Santa Cruz, CA). Functional grade anti-CD3, anti-CD28 and peridinin chlorophyll protein complex (PerCP)-labeled antibodies to CD45 were obtained from PharMingen (New York, NY). Allophycocyanin (APC)-labeled antibodies to CD105, PE-labeled antibodies to human CD73 and FITC-labeled antibodies to human CD29 were purchased from eBioscience (San Diego, CA). Unlabeled antibodies to human leukocyte antigen (HLA)-DR were obtained from Serotec (Raleigh, NC). Unlabeled pancytokeratin antibodies were obtained from Dako (Glostrup, Denmark). Neutralizing anti- transforming growth factor b receptor II (TGFβRII) antibodies were purchased from R&DSystems (Minneapolis, MN). Carboxyfluorescein diacetate succimidyl ester (CFDA-SE) was obtained from Molecular Probes (Eugene, OR). Dispase II and collagenase II (Invitrogen, Carlsbad, CA). Unless otherwise stated, all the reagents were purchased from Sigma-Aldrich (St Louis, MO).

### Mesenchymal stromal cells isolation from human limbus

Limbal stromal mesenchymal cells (L-MSC) were obtained from sclero-corneal rims from cadaveric donors. After removal the endothelium and Descemet’s membrane, the tissue was incubated with 2.4 IU/ml of dispase II for 50 min at 37 °C; afterwards, the epithelium was removed and the remaining tissue was incubated in the presence collagenase II overnight at 37 °C. The cells released from the tissue were pulled out by centrifugation and the viability was assessed by the trypan blue exclusion method obtaining up to 90% viable cells. The cells were then grown in DMEM/F12 culture medium supplemented with 10% of heat inactivated fetal bovine serum (FBS) and antibiotics (100 µg/ml streptomycin, 100 U/ml of penicillin) The L-MSC were used within 3–5 passages.

### Colony-forming unit assay

For this assay, the L-MSC were plated at two cells per cm^2^ and cultured for 10 days in a well of 6 well plate; after this period, the culture was stained with 0.5% crystal violet in methanol for 5 min and rinsed with deionized water. The colonies were manually counted. Colonies <2 mm in diameter or faintly stained were excluded.

### Population doublings assay

To determine the proliferative potential of the cells, population-doubling assay was performed. L-MSC were seeded (1.79×10^4^ cells) and counted daily during 5 days. The cell population doubling time was calculated using the Doubling Time algorithm.

### In vitro differentiation assays of L-MSC

#### Angiogenesis

To determine the capacity of these cells to differentiate into another cell linage, tubule formation assays were performed. Briefly, the cell motility and cell-cell contacts were assessed on Matrigel, a natural murine fibrosarcoma extracellular matrix (BD Biosciences, San-Jose, CA). A 96 well microplate was coated with 50 µl of Matrigel. After 1 h at 37 °C, 1×10^4^ cells in 200 µl of DMEM:F12 and recombinant human vascular endothelial growth factor (rhVEGF_165_; R&D Systems) at 0, 1, 10, and 100 µg/ml, were seeded into each well, all the assays were performed by triplicate. Culture cells were observed using an inverted microscope, and cell-cell contacts (intercellular junctions) as well as tubular reticulum formation were counted at 12 h.

#### Chondrogenesis and neurogenesis

The cells were grown in defined media: DMEM/F12, 10% FBS, 1 µmol/l insulin, 10 µg/l transforming growth factor b-1 (TGFβ1) and 300 nmol/l fresh ascorbic acid for chondrocyte differentiation; neurogenesis was induced in DMEM/F12, 10% FBS, 30 µmol/l all trans-retinoic acid. Immunodetection of nestin was used to determine the neuron phenotype, meanwhile collagen-II immunodetection was performed to identify the chondrocyte phenotype.

### Flow cytometry

Cells were washed with PBS containing 1% BSA and 0.1% sodium azide (PBA) and incubated with the indicated antibody for 15 min at 4 °C. After incubation, the cells were washed with PBA and analyzed by flow cytometry. In the cases of non-conjugated antibody, the staining was performed using a secondary flourochrome-conjugated antibody as a second step. A minimum of 1×10^4^ cells were analyzed. Flow cytometric analysis of PBMC was performed by gating first on the region corresponding to lymphocytes and lymphoblasts, followed by analysis of carboxyfluorescein diacetate succinimidyl ester (CFDA-SE) stained cells and/or antibody staining. In some assays, intracellular staining was performed as follows: after extracellular staining, the cells were fixed and permeabilized with cytofix/cytoperm kit from Becton & Dickinson (Franklin Lakes, NJ) following the manufacturer’s instructions, the intracellular staining was performed in the dark at 4 °C for 20 min, finally the cells were washed twice with PBA and acquired with a flow cytometer (FACScalibur; Becton Dickinson) and analyzed with CellQuest Software.

### Cell proliferation and suppression assays

Human peripheral blood mononuclear cells (PBMC) were obtained by Ficoll gradient. Cell viability (up to 90%) was determined by trypan blue exclusion method. Peripheral blood mononuclear cells (1×10^7^) were stained with 0.5 μM CFDA-SEM for 15 min at 37 °C in the dark and then incubated with RPMI supplemented with 10% FBS and antibiotics for additional 10 min [20]. For proliferation assays, functional grade anti-CD3 antibodies (2 μg/ml) were immobilized in a 24-flat well plate (Nunc, Rochester, NY) for 4 h at 37 °C, all experiments were performed in the presence of anti-CD28 antibodies (1 μg/ml). After 72 h of stimulation, the cells were harvested and acquired by a flow cytometer. To determine the effect of soluble factors secreted by L-MSC on suppression of T cell proliferation, L-MSC cells were placed in the upper side in a transwell system, meanwhile, the PBMC were located at the bottom of the plate. Some experiments were performed in the presence of 10µg/ml of anti-TGFβRII neutralizing antibodies. Non-stimulated cells were used as a control cells. CFDA-SE is a cell permeable dye that binds to cytoplasmic proteins producing measurable fluorescence. When stained, the cells divided by mitosis, the CFDA-SE concentration is halved between daughter cells and reductions in fluorescence detected as a measure of proliferation by flow cytometric analysis.

### Cytokine production and detection

Peripheral blood mononuclear cells 1×10^6^ were cultured in a volume of 1.0 ml complete RPMI-1640 medium in 24-well tissue plates alone or with anti-CD3/CD28 stimulation. L-MSC cells were added to these cultures at a PBMC/L-MSC ratio of 5:1. Supernatants were harvested after 72 h stimulation period. The concentrations of cytokines (interleukin-10 [IL-10] and TGBβ1) in the supernatants were measured by enzyme-linked immunosorbent assay (ELISA) using specific capture and detection mAbs purchased from R&Dsystems, and following the manufacturer’s instructions.

### Statistics

Statistical significance was determined with the GraphPad Prism Software 5.0 (GraphPad Software, Inc. La Jolla, CA), using the ANOVA test. Values were considered statistically significant when p<0.05.

## Results

### Mesenchymal stem cell population characterization

**Figure 1:**
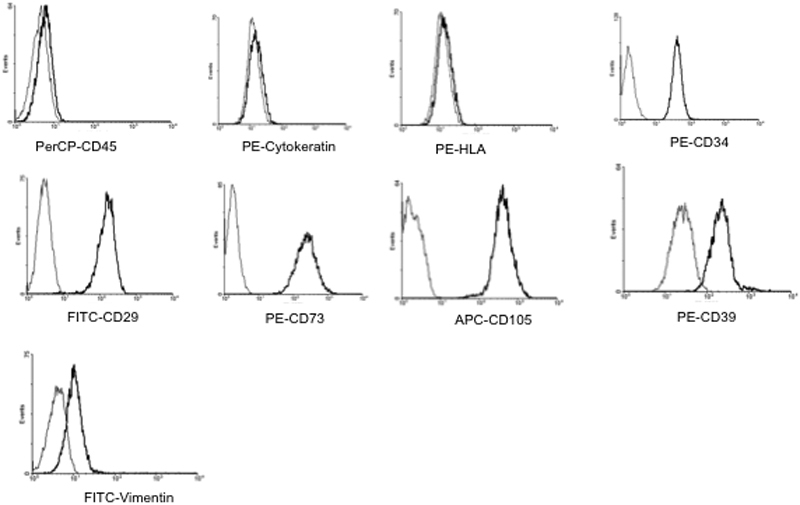
Immunophenotypical characterization of L-MSC. Cells at the 3rd passage were trypsinized, labeled with antibodies against the indicated antigens and analyzed by flow cytometry. As shown, the cells weakly expressed vimentin, meanwhile, they were positive to CD29, CD34, CD39, CD73, and CD105. On the other hand, the cells were negative to cytokeratin, HLA-DR, and CD45 expression. Representative dot plots from 3 separate samples are shown.

To identify stem cell markers on the mesenchymal stromal cells, flow cytomtery assays were performed. When analysis was made in scatter/side dot plot, a homogenous population was observed. After regionalization, the cells were analyzed by means of histograms. The Medium Flourescence Intensity (MFI) value was measured. Each result was defined by the following criteria: MFI values <10 were negative; meanwhile, MFI values ≥10 were considered positive. L-MSC population was negative to cytokeratin, CD45 and HLA-DR; indicating a non-epidermal and non-hemopoietic origin. However, these cells were positive to vimentin, CD29, CD34, CD39, CD73, and CD105, suggesting that this cell population present stromal cell markers (Figure 1). In Table 1 the results of the flow cytometric analysis are summarized.

**Table 1:** Cellular markers of mesenchymal stromal cells.

**Markers**	FC analysis*
Cytokeratin	<10
HLA-DR	<10
CD45	<10
Vimentin	12.75
CD29	181.90
CD34	54.38
CD39	409.03
CD73	323.98
CD105	508.54

*Flow cytometric analysis was defined as Medium Flourescence Intensity (MFI). Values <10 were considered to be negative.

### Colony forming unit assay

**Figure 2:**
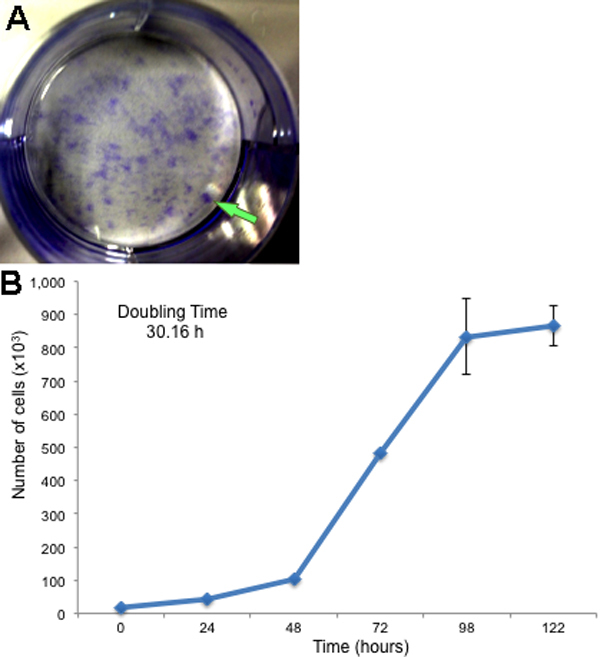
Clonogenic properties and proliferative potential of L-MSC. The cells were cultured for 10 days and stained with 0.5% crystal violet in methanol. A colony-forming unit was determined when measured >2 mm (arrow). Colonies that measured <2 mm or faintly stained were excluded (**A**). **B**: Representative growth curve of L-MSC as a function of time after the culture. Data are expressed as mean±SEM from three independent experiments.

L-MSC cultured during 10 days showed a colony forming efficiency between 30%–40% at passage 1; meanwhile, after passage 6, it was difficult to determine cell colonies, suggesting that the capability of these cells to form colonies is decreased with the respect to the number of passages (Figure 2A).

### Population doubling assay

To determine the proliferation potential of the L-MSC, a population-doubling assay was performed during five days, detailing that these cells presented a population-doubling time of 30.16 h (Figure 2B).

### Limbal mesenchymal stromal cells are capable of forming capillary like-structures

**Figure 3:**
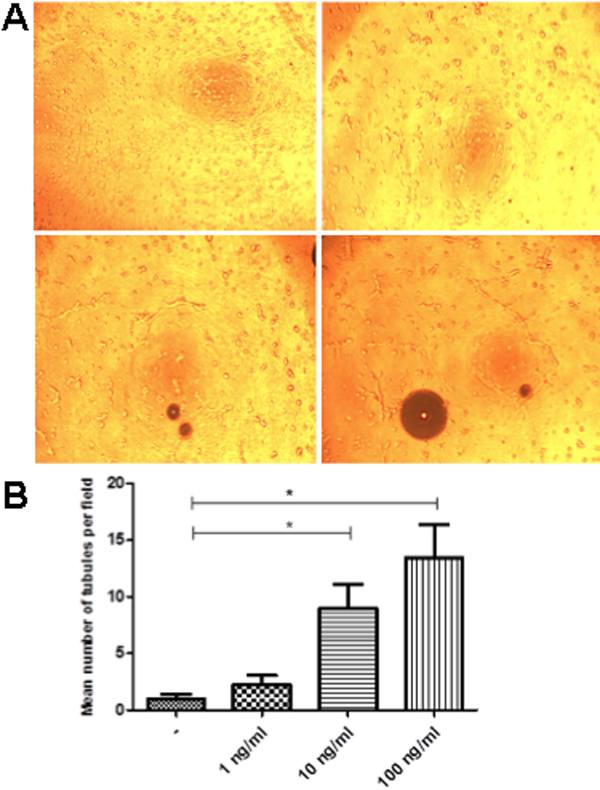
Induction of L-MSC network by VEGF. The addition of different concentrations of exogenous VEGF (0, 1, 10, and 100 ng/ml), on L-MSC seeded on matrigel significantly induced the number of cell-cell interconnections in a dose response manner (**A**). Comparison of the number of tubules formed on matrigel at different VEGF concentrations is shown in **B**. Each bar represents the mean±SEM of three independent assays (*p<0.05).

Primary culture cells from L-MSC were seeded on Matrigel. Limbal mesenchymal cells organized into tubules since 3 h of seeding on this extracellular matrix support. Network formation started, and cell-cell interaction leads to the formation of a cell network as described as reticulogenesis [21]. Interestingly, there was a dose response tubule formation when the cells were seeded in the presence of different VEGF concentrations as shown in Figure 3. These results indicate that L-MSC cells are capable of forming capillary-like structures under VEGF stimulation.

### Neurogenesis and chondrogenesis are observed in limbal mesenchymal stromal cells

**Figure 4:**
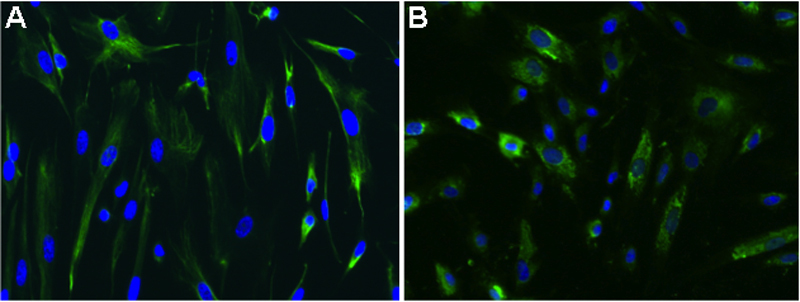
Transdifferentiated L-MSC expressed tissue specific antigens. Micrographs of immunofluorescence identifying nestin and collagen-II antigens. L-MSC were cultured in differentiation media (see materials and methods) and immunofluorescence was performed to identify nestin protein (neurogenesis) in **A**, and collagen-II (chondrogenesis) in **B**. Cells were DAPI counterstained (40×).

L-MSC were differentiated in vitro using neurogenic and chondrogenic induction media. Two weeks after the neurogenic induction, the cells showed a fibrilar positive pattern to nestin immunostaining, suggesting a neuron phenotype (Figure 4A). Similarly, these cells cultured in chondrocyte induction medium for two weeks, were positive to collagen-II immunostaining, a differentiation marker for chondrocytes (Figure 4B).

### Limbal mesenchymal stromal cells suppress lymphocyte proliferation through soluble secreted factors

**Figure 5:**
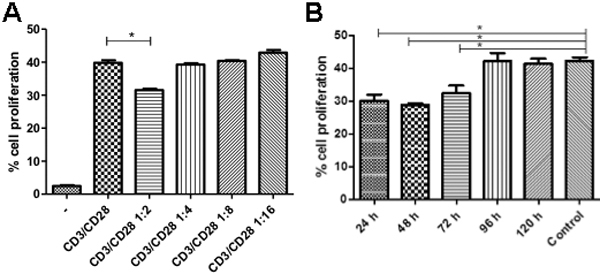
Effect of L-MSC on CD3/CD28 stimulated PBMC. **A**: CD3/CD28 stimulated PBMC were cultured alone or in indirect contact (in a transwell system) with decreasing concentrations of L-MSC (L-MSC: PBMC ratio), non-stimulated cells are also shown on left bar. **B**: CD3/CD28 stimulated PBMC were performed alone or in the presence of supernatants from L-MSC cultures. Supernatants were collected from day 1 to day 5 L-MSC cultures and added (30% v/v) to the cell cultures. Data are expressed as mean±SEM from three independent experiments (* p<0.05).

Peripheral blood mononuclear cells were stimulated through the T cell receptor (TCR; anti-CD3/CD28) in the absence or presence of L-MSC and the cell proliferation was determined by flow cytometry. To assess whether the inhibition of T lymphocyte proliferation was mediated by cell-free factors, L-MSC were separated from PBMC in tissue culture inserts with 0.4 μm membrane (Millipore, Billerica, MA). In Figure 5 is shown that the ratio 1:2 (L-MSC:PBMC) cells is the suitable proportion to induce a significant inhibition of T cell proliferation (Figure 5A). As shown in Figure 5A, cell proliferation triggered by TCR stimulation was significantly inhibited in the presence of L-MSC, compared to those cells TCR stimulated without the presence of L-MSC (p<0.05). The inhibitory effect observed in the transwell system was suggestive of soluble inhibitory factor(s). This observation was confirmed by the observation that T lymphocyte proliferation inhibition was also induced by the addition of supplemented supernatant collected from non-FBS L-MSC cultures at different times (Figure 5B).

### An immunomodulatory condition is driven by L-MSC TGFβ1 secreted cytokine on proliferating T cells

**Figure 6:**
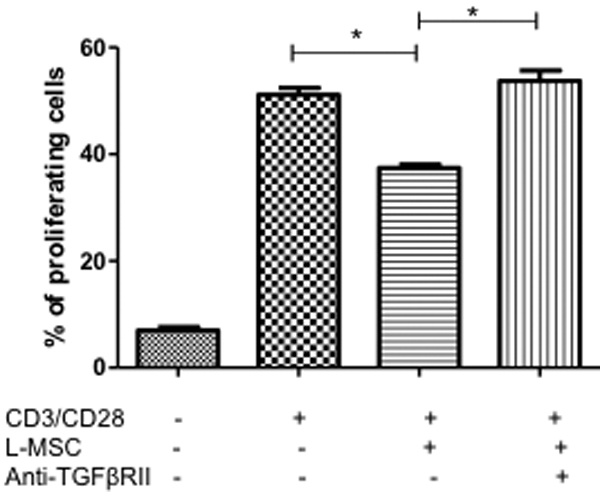
The proliferation inhibition by L-MSC was mediated by TGFβ1. PBMC were cultured stimulated with mAb anti-CD3/CD28 in the absence or presence of L-MSC. Neutralization mAb anti-TGFβRII was added to the cultures of PBMC stimulated with CD3/CD28. Each bar represents the mean±SEM from three independent experiments (* p<0.05).

To determine the possible factors that induced T cell proliferation inhibition secreted by L-MSC, IL-10 and TGFβ1 levels were determined by ELISA. IL-10 was not detectable in the supernatant from L-MSC; interestingly, TGFβ1 was constitutively expressed in the supernatant from L-MSC at high concentrations (Table 2). Interestingly, when anti-TGFβRII neutralizing antibodies were added to the antiCD3/CD28 stimulated PBMC co-cultured with L-MSC, the percentage of proliferating T cells was similar to the antiCD3/CD28 stimulated PBMC in the absence L-MSC (Figure 6).

**Table 2:** Quantification levels of IL-10 and TGFβ1on L-MSC supernatants*

**Cytokine**	**IL-10**	**TGFβ1**
Levels	ND	2000±75 pg/ml

*The cytokine levels were obtained from six independent assays.

## Discussion

In this study, we have shown that L-MSC have the capability to form tubule-like structures, as well as to differentiate into chondrocytes and neurons. Moreover, we have shown that these cells are capable to form colonies; this is an essential feature that stem cells possess [22]. In addition, these cells present immunosuppressant features by inhibiting the proliferation of T cells stimulated via T-cell receptor engagement, and this inhibition was TGFβ1 dependent. Ex vivo-expanded mesenchymal stromal cells express several non-specific markers, including CD29, CD39, CD73, and CD105 among others, meanwhile, they are negative for the expression of some hemopoietic markers, such as CD45 and HLA-DR [23]. The cell population obtained from the stromal limbus, in vitro-expanded and characterized by means of flow cytometry in this study expressed CD29, CD34, CD39, CD73, CD105, and vimentin, meanwhile, these cells were negative for CD45, HLA-DR, and cytokeratin expression. These phenotypical markers confirm the mesenchymal and non-hemopoietic origin from L-MSC. Interestingly, L-MSC population expressed both CD39 and CD73 ectoenzymes, which expression has been associated to an immunosuppressant activity [24]. Whether these molecules are directly responsible for the immunosuppressant capacity of L-MSC on T-cell proliferation, is still under study. It has been described that CD34 is a marker of hemopoietic cells, however, there are evidences that CD34 is also expressed in corneal stroma [25,26]; furthermore, Espana et al. [27] has described the constitutive expression of CD34 in cells isolated from corneal stromal tissue; in addition, it has recently shown that isolation and expansion of human stromal limbal cells expressed CD34 marker among other stem cell markers [28]; these results are in accordance with our results in which we described the presence of CD34 on this cell population; however, Dravida et al. [18] described that limbal fibroblast-like cells are negative to these marker, this difference could be associated to the ex-vivo protocols for cell expansion and derivation of limbal stromal cells.

According to the proposal of the International Society for Cellular Therapy there are three criteria to define all types of stem cells: self-renewal, multipotency and the ability to reconstitute a tissue in vivo [23]. Although, there is no specific marker for mesenchymal stem cells, the principal criteria for identification are fibroblast-like morphology, adherence to the plastic of the tissue culture flask, the prolonged capacity for proliferation and the potential to differentiate in vitro into cells of mesodermal linage [29]. Herein, we have described the ability of the cells isolated from the stromal limbus to differentiate into cells from ectodermal (neurons) and mesodermal (chondrocytes) origin; these data are similar to those obtained by Dravida et al. [18]. Furthermore, we have also shown that these cells responded to rhVEGF in a dose response manner forming tubules in a matrigel support, which means that these cells are capable to generate reticulogenesis, this observation suggests the plasticity that these limbal stem cells have in response to VEGF [30]. In addition, these cells possess stem cells characteristics such as colony forming efficiency and population doubling capacity, these results are in accordance to those reported by Polisetty et al. [4], who also described that cells obtained from human stromal limbal tissue possess these features.

Mesenchymal stromal stem cells suppress T cell responses through mechanisms not completely understood. In this study, we have shown the capacity of L-MSC to suppress proliferation of TCR activated T-lymphocytes when co-cultured in a transwell system. Similarly, T-lymphocyte proliferation suppression was also observed when cell cultures were supplemented (30% v/v) with the supernatants obtained after the culturing of L-MSC. By these two methods, we were able to determine that L-MSC secrete active factors that suppress T-cell proliferation. These results are in agreement to those obtained by other authors who described that there are soluble factors secreted by murine limbal stem cells that suppress lymphocyte proliferation [19]. With this respect, L-MSC constitutively secreted high levels of TGFβ1 when determined by ELISA; however IL-10 was not detected in the supernatant from these cells. It has been demonstrated that TGFβ1 is able to suppress T lymphocyte proliferation [31]. In this study, we could determine that inhibition of T cell proliferation appeared to be mediated by TGFβ1 secreted by L-MSC. These findings were corroborated using neutralizing antibodies against TGFβRII, which were able to re-establish the T cell proliferation. Previously, data demonstrating that blocking antibodies against transforming growth factor-1 restored bone marrow stromal cells induced suppression of T cell proliferation, confirm our findings [7]. The contribution of TGFβ1 derived from L-MSC in inducing and maintaining T regulatory cells, cannot be ruled out [32].

The ability of the L-MSC to possibly maintain a corneal phenotype and importantly, to modulate the immune response of the host, makes them an excellent candidate for generation of bioengineered corneal stromal constructs. Such constructs could be used to replace scarred stroma using partial thickness transplantation methodology currently in practice. Development of such stromal replacement constructs with L-MSC is therefore a high priority in the therapeutic application of L-MSC in humans [33].
